# What can we learn from senescent platelets, their transcriptomes and proteomes?

**DOI:** 10.1080/09537104.2023.2200838

**Published:** 2023-04-18

**Authors:** Harriet E. Allan, Ami Vadgama, Paul C. Armstrong, Timothy D. Warner

**Affiliations:** Centre for Immunobiology, Blizard Institute, Queen Mary University of London, London, UK

**Keywords:** Platelet aging, reticulated platelets, senescence

## Abstract

Research into the natural aging process of platelets has garnered much research interest in recent years, and there have long been associations drawn between the proportion of newly formed platelets in the circulation and the risk of thrombosis. However, these observations have largely been demonstrated in patient groups in which there may be underlying systemic changes that effect platelet function. Recent advances in technology have allowed in-depth analysis of differently aged platelets isolated from the peripheral blood of healthy individuals and have demonstrated that aged platelets, often referred to as senescent platelets, undergo extensive changes in the transcriptome and proteome. Ultimately, these changes result in platelets whose functions have deteriorated such that they cannot partake in hemostatic responses to the same extent as newly formed platelets. Here, we review transcriptomic and proteomic research in platelet aging in the context of health and how this research sheds light upon alterations in platelet structure and function.

## Plain Language Summary

Platelets normally last within the human circulation for approximately 10 days, however there are a number of diseases in which this life span is notably reduced. People who have platelets with such reduced lifespans have higher risks of heart attacks and strokes and reduced responsiveness to standard anti-platelet drugs. Research in recent years has aimed to understand the age-related changes that occur within platelets as they circulate in the bloodstream. In this review article, the authors provide a summary of the changes that occur during the natural aging process of platelets in healthy people, highlighting reductions in the levels of RNA and proteins. Despite these general reductions in their own RNA and proteins, research has shown that as platelets circulate they take up other RNA transcripts and proteins from the bloodstream, and this too seems a hallmark of platelet aging. The authors also discuss age-related declines in various aspects of platelet function which renders them less effective participants in the formation of blood clots.

## Introduction

Platelets are small anucleate cell fragments essential for maintaining hemostasis and preventing blood loss. Following release from the precursor megakaryocytes, platelets exist in a healthy circulation for approximately 7–10 days before their clearance in the liver or spleen.^1,2^ During their life span, platelets undergo changes in terms of their transcriptome, proteome and function.^3–6^ This means that circulating platelets exist as a heterogenous population of ages, sizes and functionalities. Dysregulation of the natural cycle of platelet formation, aging and removal has been described in a number of diseases such as diabetes mellitus, coronary artery disease and chronic kidney disease, in which there is evidence of increased platelet turnover.^7–11^ The reasons for these changes remain unknown, however evidence suggests that a shift in the age profile of the platelet population may lead to a more reactive phenotype, increasing the risk of thrombotic events and in some cases, reducing the effectiveness of anti-platelet therapies.^7,12^

Research into the fundamentals of platelet aging has gained significant research interest in recent years. By platelet aging, we refer to the aging of platelets between release from sites of production to final removal from the circulation, not differences in the platelets found in old versus young people. As our understanding of platelet aging has advanced, many terms have been coined to describe platelets at different stages of their life span. However, some of these terminologies may be misleading as they infer a change in function that has not always been fully demonstrated. Older platelets can be termed mature, senescent, non-reticulated or old platelets, whereas newly formed platelets are often referred to as immature, juvenile, reticulated or young platelets. The discrepancies in these terms mean studies investigating differently aged platelets may not always be describing the same subpopulations, potentially leading to varying results in terms of the central changes which occur during a platelet life span. This review will explore the alterations that occur during platelet aging, focussing particularly on the transcriptome and proteome and how these relate to changes in platelet function.

## Can platelets become senescent?

Platelet senescence is a term that has been adopted in recent years, however a question remains as to whether aged platelets can truly be defined as senescent? Cellular senescence was originally termed to define cells which had lost the ability to proliferate and had undergone cell cycle arrest, clearly a definition that is not relevant to platelets which are terminally differentiated and lack the ability to proliferate or undergo cell cycle arrest.^13^ However, this definition has expanded and is often used to define hallmarks of an aging cell or tissue which can be triggered by numerous factors including external stimuli, oxidative stress and mitochondrial dysfunction.^14^ If we consider senescence in this expanded sense, then old platelets can be considered to take on a senescent phenotype as recent literature has shown that there are extensive changes that occur during their continued circulation in the bloodstream.

Platelet senescence has been described in a number of studies and is in general terms a physical state that platelets adopt before their clearance by the reticuloendothelial system.^15–18^ The mechanisms governing platelet senescence are still being investigated, with two proposed processes: a “multi-hits” process and intrinsic internal timer process.^16^ The first of these suggests that platelets accumulate various damages as they circulate until they reach a threshold for their clearance.^17^ More recent research, however, has implied that platelets are released from the precursor megakaryocyte with a predefined internal molecular clock influenced by the anti- and pro-apoptotic proteins, BCL-X_L_ and Bak.^16^ While there is compelling evidence for this latter process to be more important, the ultimate trigger or triggers regulating the timing of the internal clock need to be investigated further. Further, under certain pathological conditions, the presence of exhausted platelets has been demonstrated, which may contribute to overall platelet dysfunction. Indeed, in such circumstances exhausted platelets may arise from chronic stimulation, potentially accelerating the rate of aging and thereby taking on “senescent” phenotype.^19–21^

In non-physiological settings, particularly storage of platelets for transfusion, senescence has additionally been defined by the increase in phosphatidylserine exposure and caspase-3 activation that occurs.^15^ Additionally, in transfusion bags, platelet senescence has been linked with a reduction in the platelet lipid content and a shift in the lipid profiles of phosphatidylserine, lysophosphatidylcholine and ceramide.^18^ Consistent with the generation of a senescent phenotype during platelet storage, research has shown that there is a significant increase in platelets positive for senescent-associated β-galactosidase after 3 days of storage, with almost 90% positivity by day 5.^22^ Furthermore, platelets taking on a senescent phenotype in transfusion bags have been linked with the development of platelet storage lesions. In these circumstances, it has been demonstrated that there are extensive changes in the proteome of platelet-derived extracellular vesicles which may contribute to adverse outcomes following transfusion.^23^

In summary, given their lack of nucleus and therefore proliferative ability, a “senescent” state in platelets is perhaps best used to describe an aged platelet with reduced functionality and increased clearance markers.

## Omic alterations in an aged platelet

In the current omics era, there are numerous comprehensive studies on the platelet transcriptome and proteome. The studies demonstrate approximately 9500 transcripts and over 4000 proteins are present within healthy human platelets, and that these are subject to change under various circumstances, such as following activation or in disease.^24–31^ These observations are highly suggestive that both the transcriptome and proteome are dynamic and there will be variations within an individual platelet as it circulates over time.

To assess such age-associated changes in platelets, it is necessary to be able to separate platelets on the basis of age and then study the differentiated populations. The easiest method to separate differently aged platelets would be based on size, but it is hotly contended as to whether or not platelets become smaller as they age and size isolation by centrifugation may well not represent a particularly good method of separation.^3,32–37^ A more definitive marker is ribonucleic acid (RNA) content, as it is well established that RNA declines with platelet age.^38–40^ During their biogenesis, platelets are packaged with a finite quantity of megakaryocytic RNA, which following their release into the circulation is either translated, degraded or released.^41,42^ Being anucleate, platelets are unable to transcribe new RNA and therefore RNA levels decline across platelet life span. This phenomenon has been well established and as such has allowed the monitoring of platelet turnover in patient groups through measurement of the immature platelet fractions within full blood counts.^43^

### Transcriptomic changes

Building on techniques used clinically to define newly formed platelets, a fluorescence activated cell sorting methodology has been developed in which platelets of different ages are separated based on the fluorescence of nucleic acid dyes such as thiazole orange or SYTO-13. These techniques allow for the isolation of pure populations of RNA-high young platelets and RNA-low old platelets for in-depth characterization.^3–5,44^

Accompanying the decline in RNA content across platelet life span, Bongiovanni *et al*. and Hille *et al*. have shown that there are also changes in the platelet transcriptome.^3,4^ These studies demonstrate that following fluorescence activated cell sorting based on either thiazole orange or SYTO-13 staining, respectively, the RNA content of those defined as newly formed platelets (top 10–20% of thiazole orange/SYTO-13^bright^) is 0.88–1.2fg per platelet decreasing to 0.35–0.4fg per platelet in the aged subpopulation of platelets (bottom 20–30% thiazole orange/SYTO-13^dim^).^3,4^ In these transcriptomic studies, Bongiovanni *et al*. demonstrated that aged platelets had higher levels of 670 transcripts and lower levels of 1074 transcripts compared to newly formed platelets. Similarly, Hille *et al*. identified higher levels of 1264 transcripts and lower levels of 1212 transcripts in aged platelets compared to newly formed platelets. As there is only around half the quantity of RNA per platelet in the older platelets compared to young platelets, it is surprising to see higher levels of so many transcripts in this subpopulation.

From these analyses, it is unclear whether these changes are indicative of changes in the absolute transcript levels or are relative to the general declining RNA content. However, relative increases in RNA transcripts should not be surprising as it has been established that platelets can act as sponges, endocytosing molecules from the circulation, therefore some of these transcripts may originate from non-platelet origins.^32^ For example, Clancy *et al*. demonstrate that following *in vitro* incubation with endothelial cells for 2 hours, the levels of 18 transcripts increased in platelets suggesting active uptake of RNA.^32^

Similarly, the rate of loss of each transcript may not be equal and therefore lead to an increase in relative abundance between ages. Evaluation of a small number of transcripts isolated from temporally labeled mouse platelets showed the same decline in RNA content as platelets age.^6^ However, the rate of decline varied amongst cellular compartments, with granule associated transcripts having the slowest reduction rate, whilst signaling and receptor transcripts displayed the fastest rates of decline. This data is indicative of selective retention of transcript, potentially to favor the preservation of particular functions.^6^

Amongst the transcripts detectable at higher levels in newly formed platelets are surface receptors important for hemostatic responses including, glycoproteins (GP)V and VI, integrin alpha IIb (Itga2b) and integrin beta-3 (Itgb3), the thrombin receptor PAR-4, as well as the transcripts involved in calcium homeostasis, including stromal interaction molecule 1 (STIM1) and calcium release-activated calcium modulator 2 & 3 (Orai2, Orai3). Furthermore, higher levels of cytoskeletal transcripts, such as filamin A and talin 1 were detectable in newly formed platelets.^3,4^ On the other hand, these studies highlight higher levels of transcripts for complement C5 and interleukin 7 in the aged platelets. The large list of transcripts which were higher in aged platelets are involved in numerous processes including RNA binding and export, ribosome biogenesis, cell division and apoptotic regulation. In addition, Hille *et al*. demonstrated that aged platelets had higher levels of transcripts associated with other circulatory cells including hemoglobin (HBA1, HBA2, HBB), indicative of the platelet’s ability to endocytose molecules from the circulation.^3^

Despite contradictory literature on changes in size with platelet age, if we accept for discussion that newly formed platelets are larger and aged platelets are smaller, a transcriptomic study on these subpopulations identified 2314 transcripts that are unique to “older” platelets and 378 that are unique to “young” platelets.^32^ The authors of this study note that these results were contrary to their anticipated results with the data demonstrating a more varied transcriptomic profile in “older,” small platelets. However, in this study, the changes highlighted using transcriptomics were not substantiated using real-time quantitative polymerase chain reaction (RT-qPCR), with the coverage tracks for these genes showing distinctive coverage patterns between the two subpopulations. Furthermore, the authors highlight that the RNA isolated from the “old,” smaller platelets was consistently of a lesser quality than the “young,” larger platelets which may provide an explanation as to why the transcriptomic data could not be validated with RT-qPCR.^32^

It is clear that platelet aging is associated with significant alterations in the transcriptome, however given that platelets lose over half their RNA across their life span, it remains unclear how there could be increases in such a large number of transcripts in old platelets. As platelets lack a nucleus, they do not have the capacity to generate new RNA and therefore only have a finite repertoire of RNA inherited from the precursor megakaryocyte. It is, however, well established that platelets have the ability to endocytose molecules from the circulation, which may account for some of these increases.

### Proteomic changes

Platelets are equipped with the machinery required for protein synthesis, however their translational capacity is thought to be low.^45,46^ To our knowledge, only one comprehensive proteomic study has been performed on differently aged platelets separated by nucleic acid dye staining and cell sorting. This proteomic analysis, performed in our laboratory, revealed that platelet aging is associated with a reduction in protein content from 8.7 ± 2.6 pg per platelet in newly formed platelets to 4.7 ± 1.2 pg per platelet in aged platelets.^5^ Despite this general decline in protein, there were alterations in particular protein levels. Firstly, supporting changes at the transcriptomic level, aged platelets have a reduction in proteins involved in hemostatic responses including integrin alpha IIb and integrin beta-3, P-selectin as well as STIM1.^47^

Against a background of protein loss, a number of cytoskeletal-associated proteins such as Emerin, Gelsolin and Twinfilin-2 were lost to a greater extent.^5^ This accelerated loss of cytoskeletal proteins is of particular interest as the cytoskeleton is fundamental during platelet activation and its rearrangement underpins all stages of adhesion and aggregation. Furthermore, a platelet-specific knockout mouse model of Twinfilin-2a showed an increase in platelet turnover, suggesting that the cytoskeleton and associated proteins are important in determining platelet life span.^48^ In addition, aged platelets also have a significant reduction in mitochondrial-associated proteins, suggesting that platelet aging may be accompanied by changes in metabolism.^5^ Consistent with a loss of organelles in aged platelets, a number of proteins traditionally associated with the endoplasmic reticulum and Golgi apparatus including Reticulon-1, Inverted Formin-2 and Extended Synaptotagmin-1 were found to be reduced in aged platelets, indicating their inheritance from precursor megakaryocytes as these structures are largely lacking from platelets.^5^

Although most proteins detected as differentially expressed between young and old platelets had higher levels in the newly formed subpopulation, proteomics revealed a small group of proteins that had relatively higher levels in the old platelets. These comprised of a number of proteins from other vascular cell origins including Haemoglobin (HBA) and Complement proteins (C4A, CO5, C8G) indicative of active endocytic pathways during platelet aging.^5^

### Pathway analysis

**Figure 1.**
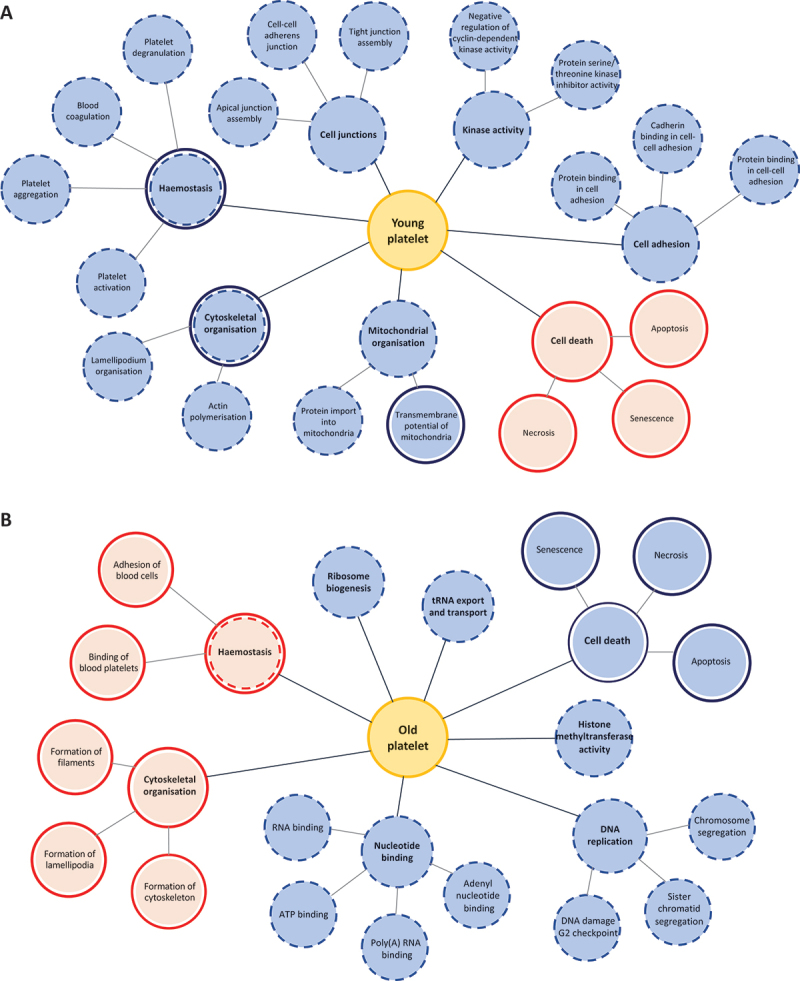
Predicted changes in biological processes in differently aged platelets. Summary of pathway analysis and gene ontology terms predicted to be affected by changes in the transcriptome and proteome in (a) young platelets and (b) old platelets.^3–5^ The blue indicates a predicted increase, and the red indicates a predicted decrease in the biological process, with the dashed lines indicating changes at the transcriptomic level and solid lines indicating changes at the proteomic level.

Gene ontology and pathway analysis of the transcriptomic and proteomic data indicated that these changes across platelet life span may affect numerous biological processes (Figure 1). Newly formed platelets have a predicted enhancement in hemostatic pathways, including platelet activation, aggregation and blood coagulation. In addition, cytoskeletal organization, mitochondrial organization, cell adhesion, cell junction formation and kinase activity were also predicted to be enhanced in young platelets. Conversely, cell death pathways including necrosis, apoptosis and senescence were predicted as down regulated pathways in newly formed platelets (Figure 1a).^3–5^

Consistent with these data, aged platelets had a predicted downregulation in hemostatic pathways as well as cytoskeletal organization. On the other hand, old platelets were shown to have an enhancement in cell death pathways, indicative of their aged statuses. Furthermore, older platelets have a predicted enhancement in pathways associated with DNA replication, ribosome biogenesis and nucleotide binding, suggesting potential changes in the translational capacity with platelet aging (Figure 1b).^3–5^

## The link between omics and platelet structure

Analysis of the platelet transcriptome and proteome provided novel insights into alterations which may be appearing at the structural level. Reductions in cytoskeletal-associated transcripts and proteins encouraged further investigation using immunofluorescence and transmission electron microscopy (TEM). Immunofluorescence analysis demonstrated that the cytoskeleton of an aged platelets is less dense and complex compared to newly formed platelets.^5^ Contradictory to this, TEM indicated no differences in percentage of platelets with visible microtubule structures between the young and old platelets.^3^ TEM, confocal microscopy and flow cytometry showed that aged platelets have approximately half the number of mitochondria of newly formed platelets, suggesting age-related reductions in platelet metabolic activity.^3,5,49,50^ Furthermore, the number of α-granules and open canalicular system openings were significantly reduced in aged platelets compared to young platelets.^3^ Whilst there is still contradictory evidence regarding changes in size as platelets age, it is clear that there are extensive alterations in the intracellular structure, which may cause alterations in the overall buoyant density of the platelet, a phenomenon originally described in the 1980s.^37^

## The link between omics and function during platelet aging

The pathway analyses performed on the transcriptomic and proteomic data also suggested changes in a number of biological process, raising the question as to whether aged platelets are functionally different from newly formed platelets. As noted above, research over the past few decades has highlighted an association between an increased proportion of young platelets, indicated by the immature platelet fraction, and an overall enhancement in platelet reactivity.^51^ However, these studies have largely been performed in patient groups with altered platelet turnover, therefore they may not be a true representation of the function of young and old platelets produced under healthy, steady state conditions.

Consistent with the predicted alterations in biological processes, in-depth characterization of the functions of differently aged platelets confirmed that newly formed platelets are more haemostatically reactive than aged platelets.^3,5^ Interestingly, aged platelets are present in a lower proportion of aggregates *in vitro*, generally bind to the periphery, with the newly formed platelets forming the core.^5^ Furthermore, given the changes in the cytoskeleton, aged platelets have an impaired ability to adhere and spread on fibrinogen.^5^ Consistent with a diminished aggregatory and adhesion response, aged platelets have reduced calcium flux in response to physiological stimulation as well as ionomycin, indicating calcium stores may diminish with platelet age.^5,6^ Further confirmation of this reduced activation capacity has come from recent research showing that aged platelets express lower levels of GPVI, indicating a reduced ability for collagen activation and signaling.^6,33^ In addition, aged platelets have a reduced capacity to potentiate the activation response through granule secretion and eicosanoid synthesis.^3–5,33^ These *in vitro* experiments confirm the alterations in platelet function previously described in patient groups, with newly formed platelets being the most reactive and driving the hemostatic response.

## Conclusions

**Figure 2.**
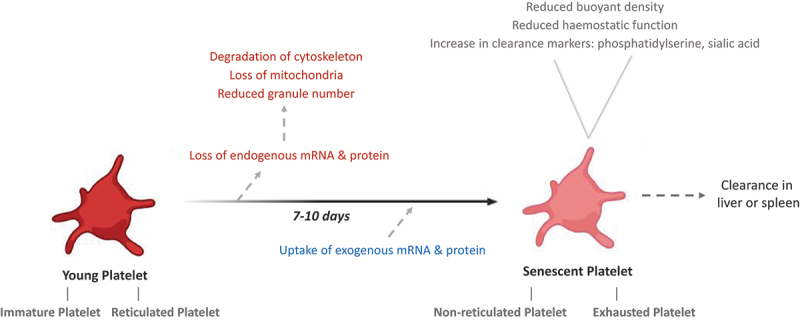
Summary of the changes that occur during a platelet’s life span in the healthy circulation. Following release from the precursor megakaryocyte, platelets circulate for 7–10 days during which they undergo extensive changes. Platelet aging over this period is associated with a reduction in endogenous RNA and protein content, leading to a reduced hemostatic function. However, platelets also have the capability to endocytose exogenous RNA and protein which may affect their function in ways that have yet to be defined.

Differently aged platelets are often referred to as separate and isolated subpopulations, rather than platelets at either ends of a continuum with the changes in the transcriptome, proteome and functionality as platelets age highlighting this continuum. However, to best describe the physical attributes of an aged “senescent” platelet, these studies highlight a platelet with reduced RNA and protein content, loss of structural integrity and reduced hemostatic capabilities (Figure 2). In essence, as platelets age they become fragile and lose the ability to carry out their primary function, hemostasis; this deterioration could be described as platelets taking on a senescent phenotype.

The processes determining platelet life span still remain unclear; however, the data presented in this review highlight a possibility that this general decline may reach a threshold in which aged platelets are no longer viable and therefore marked for clearance. These omic studies provide novel insights into the fundamental changes occurring during platelet aging, advancing our understanding of potential determinants of platelet life span. Functional characterization supporting the omic data confirms the dogma that aged platelets are less reactive than newly formed platelets. Therefore, a shift in the age profile of platelets toward a population with a higher proportion of newly formed platelets will indeed lead to a more reactive total platelet population and potentially an increased risk of thrombotic events.

Given the advancement in technologies, we are at a stage where we will be able to begin exploring the differences between young and senescent platelets in greater depth. Investigating more subtle changes in post-translational modifications will allow better understanding of particular signaling pathways which may be altered throughout a platelet’s life span. It is well documented that platelets have alternative functions aside from hemostasis, such as participating in inflammation and immunity, with further work and omic studies, we may be able to explore these different functions throughout platelet life span. In conclusion, the question “What can we learn from senescent platelets, their transcriptomes and the proteomes?” is an interesting one, and one that we are becoming increasingly well equipped to answer.
